# Administration of a Polyphenol-Enriched Feed to Farmed Sea Bass (*Dicentrarchus labrax* L.) Modulates Intestinal and Spleen Immune Responses

**DOI:** 10.1155/2016/2827567

**Published:** 2015-12-08

**Authors:** Thea Magrone, Sergio Fontana, Flavia Laforgia, Teresa Dragone, Emilio Jirillo, Letizia Passantino

**Affiliations:** ^1^Department of Basic Medical Sciences, Neuroscience and Sensory Organs, University of Bari, 70124 Bari, Italy; ^2^Farmalabor Srl, 76012 Canosa di Puglia, Italy; ^3^Department of Emergency and Organ Transplantation, University of Bari, 70124 Bari, Italy

## Abstract

Farmed fish are exposed to a continuous antigenic pressure by microbial and environmental agents, which may lead to a condition of chronic inflammation. In view of the notion that polyphenols, largely contained in fruits and vegetables, are endowed with antioxidant and anti-inflammatory activities, farmed sea bass (*Dicentrarchus labrax* L.) have been administered with red grape polyphenol-enriched feed. Polyphenols were extracted from the seeds of *Canosina* Nero di Troia *Vitis vinifera* and mixed with conventional feed at two different concentrations (100 and 200 mg/kg, resp.). Fish samples collected at days 223 and 273, respectively, were evaluated for intestinal and spleen cytokine release as well as for spleen macrophage (MØ) and melanomacrophage center (MMC) areas and distribution. Data will show that in treated fish decrease of intestinal interleukin- (IL-) 1*β* and IL-6 and increase of splenic interferon- (IFN-) *γ* occur. On the other hand, in the spleen reduction of MØ number seems to parallel increase in MMCs. Collectively, these data suggest that polyphenol-administered sea bass generate lower levels of intestinal proinflammatory cytokines, while producing larger amounts of spleen IFN-*γ*, as an expression of a robust and protective adaptive immune response. Increase of MMCs corroborates the evidence for a protective spleen response induced by feed enriched with polyphenols.

## 1. Introduction

It is well known that fish immune cells mainly include lymphocytes, dendritic cells, monocytes, macrophages, granulocytes, and thrombocytes [1]. In teleost fish, major lymph reticular tissues are head kidney, spleen, thymus, liver, and mucosa-associated lymphoid tissues as in the gut [2]. Accordingly, these tissues may represent the primitive analogues of fish lymph node germinal centers, even if their morphology and immune function are still under investigation [3–6].

In fish, melanomacrophage centers (MMCs) are defined as a group of pigmented macrophages (MØ) under form of a nodular cluster of MØ characterized by heterogeneous inclusions such as degradation products of cells [7]. MMCs are devoted to the destruction and recycling of exogenous and endogenous antigens [8], even including storage of iron as a consequence of erythrophagocytosis [9]. All these processes generate cell debris, melanin pigments, hemosiderin granules, and lipofuscin residues [10], as well as lipid droplets, basic protein aggregates, and neutral mucopolysaccharides [11]. Moreover, some types of intracellular granules contain trace metals [12–15]. The pigment deposits contained in vacuoles suggest a mechanism of phagocytosis by MMCs as an expression of early antimicrobial defenses [16–18].

Number, size, and pigment distribution of MMCs depend on fish species [19, 20], organs [11, 21, 22], age [23–25], sexual activity [26, 27], nutritional status, and fish health [10, 28–31].

The growth of aquaculture, associated with the intensification of production systems, has increased the demand for high-quality feedstuff in order to improve fish health without any side effects for consumers [32, 33]. Fish meal has traditionally been used as the main feed ingredient in preparation of fish feed, due to its high protein content and balanced amino acid profile. Because of its recent shortage in global production, coupled with increased demand and competition for its use in livestock and poultry feeds, prices of fish meal have become unaffordable [34]. Therefore, sustainable aquaculture depends on a perfect balance between growth and healthy conditions of fish. The use of antibiotics and chemotherapeutics to combat fish infections may generate resistant pathogens, bioaccumulation, and environmental pollution. Furthermore, commercial vaccines are expensive for fish farming practices and must be specific against particular pathogens [35].

Nowadays, use of plant proteins to replace fish meal without reducing the performance has started. The administration of probiotics and prebiotics to fish seems to favor the growth of a protective microbiota [36, 37], which, for its immunomodulating activities, may represent a very promising biological control for aquaculture. Therefore, investigations of spleen MMCs have provided useful knowledge on the fish health status also in relation to the type of nutrition [38–43].

Among natural products, polyphenols, largely present in fruits and vegetables, have been shown to scavenge oxygen and nitrogen derived free radicals, modulating antioxidant enzymes and cellular redox transcription factors [44]. In particular, the protective effects of polyphenols consist in the continuous removal of various reactive oxygen species from cells, such as singlet oxygen, peroxynitrite, and hydrogen peroxide in order to maintain healthy metabolic functions [45]. They may also affect cell-to-cell signaling, receptor sensitivity, inflammatory enzyme activity, or gene regulation [46, 47]. According to our own studies in both animal models and humans polyphenols from red grapes are endowed with antioxidant and anti-inflammatory activities, also keeping in equilibrium the immune machinery [48, 49].

A few studies have been conducted on the use of polyphenols in farmed fish in order to evaluate their antioxidant and anti-inflammatory effects. One study investigated the* in vitro* effects of resveratrol, mangiferin, and (–)-epigallocatechin-3-gallate on the histiophagous ciliate* Philasterides dicentrarchi*, which causes fatal scuticociliatosis in farmed turbot* Scophthalmus maximus* L. Of the 3 polyphenols, resveratrol showed strongest antiprotozoal activity, reducing ciliate density after 1-week culture. In view of these findings, the potential utility of chemotherapy with polyphenols as a strategy for the control of scuticociliatosis in farmed turbot has been emphasized [50].

In another research, the beneficial effects of polyphenols derived from waste water from an olive mill, obtained by nonplastic molecular imprinting device, were evaluated in a hypercholesterolemic diet on* Carassius auratus*, commonly known as goldfish, that was selected as experimental model. Results show the beneficial activity of polyphenols with a reduction of the damage in the steatotic group, confirming that they may be used for the treatment of diseases by lipid accumulation in feed of farmed fish. This dietary approach may improve the organoleptic and nutritional quality. Finally, the beneficial effects of waste oil extract should be suggested in the context of research programs focused on the products to the health system [51].

In the present study, in farmed sea bass (*Dicentrarchus labrax* L.) the effects of a diet rich in polyphenols on the cytokine release from gut and spleen tissues as well as deposition of MMCs in spleen were evaluated.

## 2. Materials and Methods

### 2.1. Polyphenol Extracts

*Canosina* red grape from Nero di Troia is an autochthonous* Vitis vinifera* grape cultivar which grows in Apulia (South Italy). It is characterized by thick skinned and small sized berries. Frozen seeds from berries were extracted by percolation using ethanol/water (70 : 30). Then, the extract was first analyzed by means of liquid chromatography with diode array detection to define the polyphenol composition. Thereafter, the extract was purified on a synthetic adsorbent brominated resin and percentage of polyphenol content was determined.

The extracts were evaluated for their potential antioxidant effects by using the 2,2-diphenyl-1-picrylhydrazyl assay which measures the ability of test agents to scavenge radicals [52].

### 2.2. Preparation of Feed and Dietary Regimen

Fish diet consisted in conventional feed mixed with two different concentrations of grape extracts, administered 3-4 times a day:

(1) Fish (*n* = 30) fed mix composed of 50 g of grape extracts in 5 kg of cornstarch, namely, 10 g of mix (100 mg of extract) for 1 kg of conventional pellet (100 mg/kg).
(2) Fish (*n* = 30) fed mix composed of 100 g of grape extracts in 5 kg of cornstarch, namely, 10 g of mix (200 mg of extract) for 1 kg of conventional pellet (200 mg/kg). For preliminary experiments, lower concentration of grape extracts, for example, 1, 10, and 50 mg/kg, was not effective in our test system.

Controls (*n* = 30) were represented by fish fed conventional feed.

### 2.3. Sample Collection

Spleen samples were collected from a total of 90 immature (juvenile) samples of sea bass reared in captivity in a farm near Lesina lake (Foggia, Italy). Sample collection took place during winter at 223 days (T1) and 273 days (T2) from the beginning of the experiment.

Fish, reared in experimental conditions, were treated according to the “Council Directive 86/609 EEC for the protection of animals used for experimental and other scientific purposes” and the “Ethical Justification for the Use and Treatment of Fishes in Research” [53].

### 2.4. Immunological Investigations

Specimens were taken from spleen and both pyloric (P) and terminal (T) gut (G) segments of treated and untreated fish, respectively.

Gut portions were placed in Petri dishes containing RPMI 1640 (Miltenyi Biotec, Bergisch Gladbach, GE) plus streptomycin (100 mg/mL) (Biowhittaker, Walkersville, USA) and 1% penicillin (Biowhittaker) and sliced with scissors to obtain GP and GT samples, respectively. Both segments were then incubated in Petri dishes containing RPMI 1640 for 2 and 24 h at 14°C, respectively. Afterwards, supernatants of GP and GT cultures were obtained by centrifugation at 10,000 ×g for 10 min at 4°C and stored at −30°C, until use.

Spleen specimens were placed in Petri dishes containing RPMI 1640 plus 0.2% heparin and passed through a cell strainer with a 70 *μ*m nylon membrane (Becton Dickinson, Bedford, MA), gently forced with a 1 mL syringe plunger, and filtered in medium, to yield a single cellular suspension for each case. After incubation for 2 and 24 h at 14°C, respectively, 1 mL of cell cultures was put into Eppendorf cups and centrifuged at 10,000 ×g for 10′ at 4°C. Finally, culture supernatants were collected and stored at −30°C, until use [54].

Concentrations (pg/mL) of fish interleukin- (IL-) 1*β*, IL-6, and interferon- (IFN-) *γ* in supernatants were determined by specific ELISA kits (Cusabio Biotech Co., Wuhan, Hubei, China) according to manufacturer's instructions. Cytokine concentrations were read at 550 nm by means of an ELISA reader (iMark Microplate Absorbance Reader, BioRad, Hercules, California, USA). Concentrations obtained for each cytokine were multiplied by the dilution factor to obtain sample values.

### 2.5. Basic Histology and Histochemistry

All fishes were anaesthetized with Tricaine 1 : 5000 (Fluka BioChemika, Buchs, Switzerland) according to the guidelines for Euthanasia of Nondomestic Animals American Association of Zoo Veterinarians (2006). GP, GT, and spleen of each fish were removed, fixed in 10% buffered formalin, later washed in running water, dehydrated in increasing ethanol concentrations, and embedded in paraffin wax.

Sections of tissue (5 *μ*m thick) were processed for the following: (a) Hematoxylin-Eosin (H&E) staining (Merck, Darmstadt, Germany); (b) Perls-Van Gieson staining (Bio-Optica, Milan, Italy) to identify ferric iron; (c) Mallory staining (Merck, Darmstadt, Germany) to detect lipofuscin pigments and ceroids. The identification of MØ was performed using the *α*-Naphthyl Acetate Esterase (Anae) (Sigma Diagnostics, St. Louis, MO, USA) and Peroxidase (Perox) (Sigma Diagnostics) methods.

### 2.6. Quantification of Melanomacrophage Centers

The surface occupied by MØ and MMC (*μ*m^2^ spleen parenchyma) was counted and measured randomly for a number of 100 elements, selected at digital fields. Each digital field was photographed with a 40x objective with a digital camera (XC-003P, Sony, Tokyo, Japan) connected to a light microscope (Laborlux 12, Leitz, Wetzlar, Germany). Measurements were performed using an image analysis software (QWin, Leica, Cambridge, England).

### 2.7. Statistical Studies

Statistical differences for concentrations of cytokines from gut and spleen tissues after 2 and 24 h incubation, respectively, between untreated and treated samples, both at T1 and T2, and number and surface occupied by MØ and MMCs were compared. Statistical analysis was performed using the GraphPad Prism statistical software release 5.0 for Windows Vista. Bonferroni's test was used for comparison between controls and treated samples. Statistical significance was set at *P* < 0.05.

## 3. Results

As far as the polyphenol content of the red grape extracts is concerned percentages were the following: proanthocyanidins (101.8%) and catechins plus epicatechin (10.37%).

**Figure 1:**
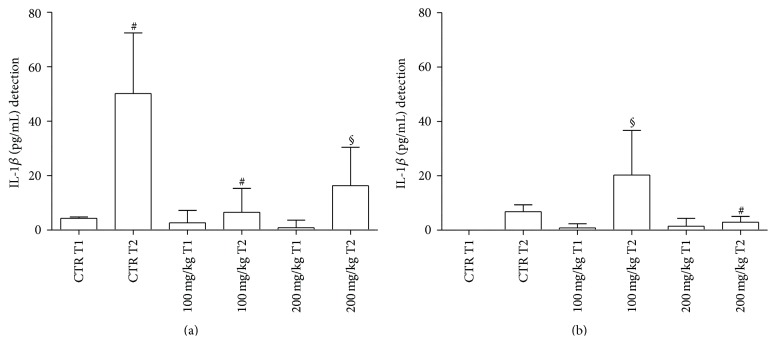
Fish IL-1*β* levels from GP (a) and GT (b) supernatants after 2 h incubation. CTR = untreated samples; T1 = before treatment; T2 = at the end of treatment. Statistical analysis was performed using the GraphPad Prism statistical software release 5.0 for Windows Vista. Bonferroni's test was used for comparison between the controls and treated samples at both concentrations. Statistical significance was set at *P* < 0.05. (a) ^#^ *P* < 0.0001 CTR T1 versus CTR T2; ^#^ *P* < 0.0001 CTR T2 versus 100 mg/kg T2; ^§^ *P* < 0.01 CTR T2 versus 200 mg/kg T2. (b) ^§^ *P* < 0.01 100 mg/kg T1 versus 100 mg/kg T2; ^*∗*^ *P* < 0.05 100 mg/kg T2 versus 200 mg/kg T2.

*(a) Cytokine Release from Gut and Spleen following 2 h Incubation.* With regard to GP supernatants, concentrations of IL-1*β* are represented in Figure 1(a). At T2, in treated samples amounts of IL-1*β* were significantly lower than those observed in the respective controls. No differences were noted in relation to polyphenol concentrations used.

Results related to determination of IL-1*β* in GT supernatants are expressed in Figure 1(b). A significant increase in IL-1*β* was observed at T2 with 100 mg/kg polyphenol dose in comparison to other treated samples.

With regard to spleen IL-1*β* production, no significant differences between the various samples were detected (data not shown).

**Figure 2:**
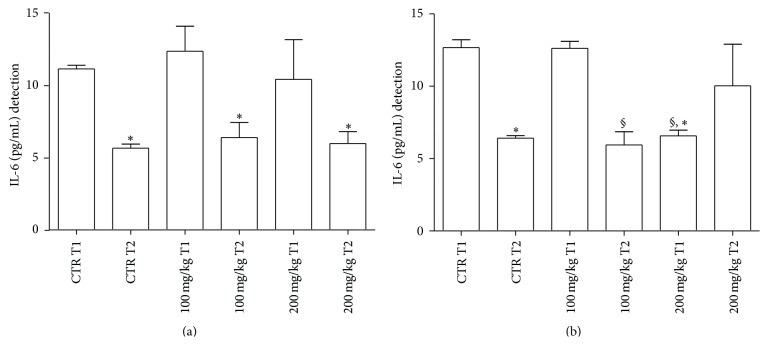
IL-6 levels released from GP (a) and GT (b) supernatants after 2 h incubation. For abbreviations and statistical analysis see Figure 1. (a) ^*∗*^ *P* < 0.05 CTR T1 versus CTR T2; ^*∗*^ *P* < 0.05 100 mg/kg T1 versus 100 mg/kg T2; ^*∗*^ *P* < 0.05 200 mg/kg T1 versus 200 mg/kg T2. (b) ^*∗*^ *P* < 0.05 CTR T1 versus CTR T2; ^*∗*^ *P* < 0.05 CTR T1 versus 200 mg/kg T1; ^§^ *P* < 0.001 100 mg/kg T1 versus 100 mg/kg T2; ^§^ *P* < 0.001 100 mg/kg T1 versus 200 mg/kg T1.

Determination of IL-6 in the GP supernatants is represented in Figure 2(a). In untreated samples, an increase in IL-6 levels was noted at T1 in comparison to T2. In treated samples with both concentrations of polyphenols levels of IL-6 decreased in a statistically significant manner at T2 versus T1. However, values of these treated samples were not significantly different from those of controls at the same time points.

With special reference to GT supernatants, IL-6 determination is depicted in Figure 2(b). In untreated samples, an increase in IL-6 levels was noted at T1 in comparison to T2. In the case of treated samples, T2 values of IL-6 were lower than T1 levels using 100 mg/kg of polyphenols. However, T1 values with 200 mg/kg were lower than the respective untreated counterpart.

With regard to spleen IL-6 production, no significant differences between the various samples were observed (data not shown).

**Figure 3:**
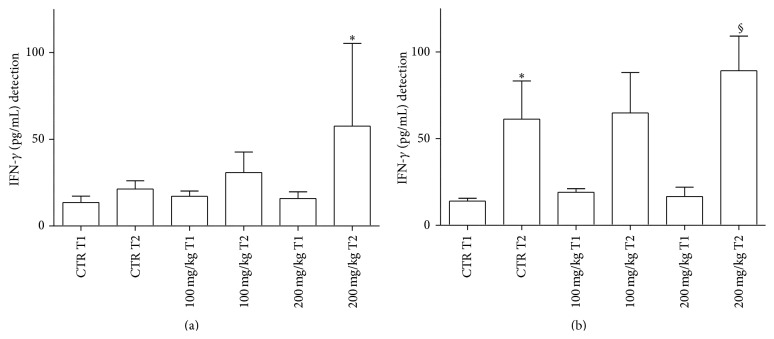
IFN-*γ* levels released from GT (a) and spleen (b) supernatants after 2 h incubation. For abbreviations and statistical analysis see Figure 1. (a) ^*∗*^ *P* < 0.05 200 mg/kg T1 versus 200 mg/kg T2. (b) ^*∗*^ *P* < 0.05 CTR T1 versus CTR T2; ^§^ *P* < 0.01 200 mg/kg T1 versus 200 mg/kg T2.

In the GP supernatants, IFN-*γ* levels decreased at T1 in treated samples with 200 mg/kg dose in comparison to T2 controls (data not shown). In Figure 3(a), at T2, levels of IFN-*γ* released from GT were higher with 200 mg/kg dose in comparison to the same concentration at T1.

At T2, spleen IFN-*γ* release significantly increased in all samples considered (Figure 3(b)). However, at T2, levels of IFN-*γ* in fish treated with 200 mg/kg were significantly higher than those observed at T1 in both treated and untreated samples.

*(b) Cytokine Release from Gut and Spleen following 24 h Incubation*. In GP supernatants, at T2, IL-1*β* concentrations of untreated samples were higher in comparison to 200 mg/kg treated samples at T1 (data not shown).

In GT supernatants, no differences in terms of IL-1*β* concentrations were noted in all samples (data not shown).

Values of spleen IL-1*β* production were not statistically significant in all samples (data not shown).

**Figure 4:**
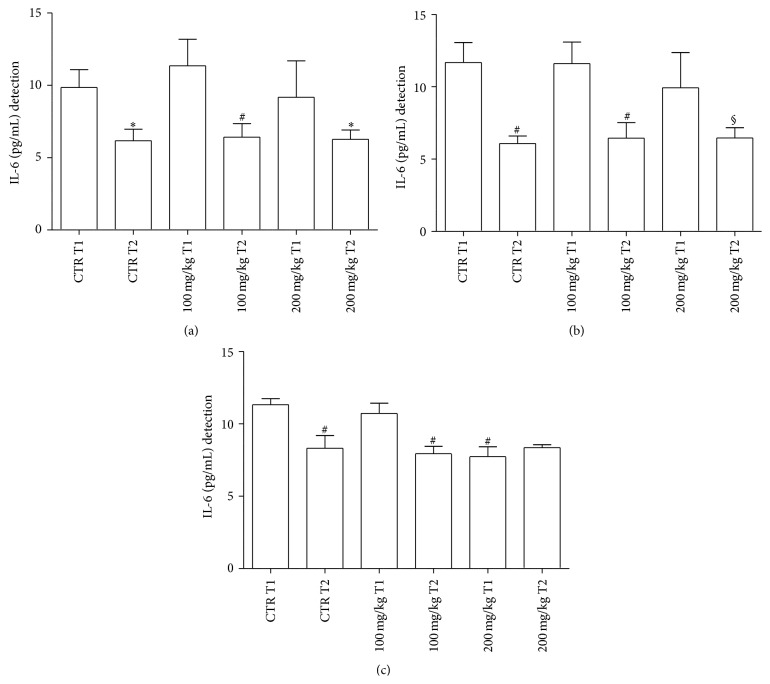
IL-6 levels released from GP (a), GT (b), and spleen (c) supernatants after 24 h incubation. For abbreviations and statistical analysis see Figure 1. (a) ^*∗*^ *P* < 0.05 CTR T1 versus CTR T2; ^§^ *P* < 0.01 200 mg/kg T1 versus 200 mg/kg T2. (b) ^#^ *P* < 0.0001 CTR T1 versus CTR T2; ^#^ *P* < 0.0001 100 mg/kg T1 versus 100 mg/kg T2; ^§^ *P* < 0.01 200 mg/kg T1 versus 200 mg/kg T2. (c) ^#^ *P* < 0.0001 CTR T1 versus CTR T2; ^#^ *P* < 0.0001 CTR T1 versus 200 mg/kg T1; ^#^ *P* < 0.0001 100 mg/kg T1 versus 100 mg/kg T2; ^#^ *P* < 0.0001 100 mg/kg T1 versus 200 mg/kg T1.

In GP supernatants, IL-6 amounts were significantly lower at T2 versus T1 in all samples considered (Figure 4(a)). However, IL-6 values of treated samples were not significantly different when compared to controls.

In GT supernatants, the same pattern of response was observed in terms of a significant reduction of T2 versus T1 values in all samples (Figure 4(b)).

Spleen IL-6 levels in untreated and treated samples were significantly higher at T1 versus T2. In the spleen, T1 values of IL-6 with 200 mg/Kg were lower than the respective untreated counterpart (Figure 4(c)).

In GP supernatants, IFN-*γ* production was basically the same in all samples (data not shown).

**Figure 5:**
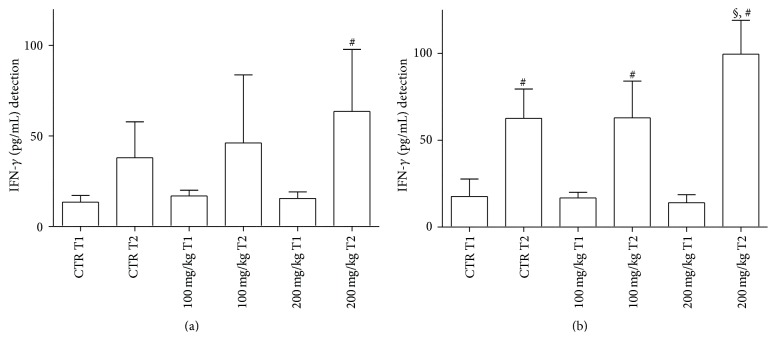
IFN-*γ* levels released from GT (a) and spleen (b) supernatants after 24 h incubation. For abbreviations and statistical analysis see Figure 1. (a) ^#^ *P* < 0.0001 200 mg/kg T1 versus 200 mg/kg T2; (b) ^#^ *P* < 0.0001 CTR T1 versus CTR T2; ^§^ *P* < 0.01 CTR T2 versus 200 mg/kg T2; ^#^ *P* < 0.0001 100 mg/kg T1 versus 100 mg/kg T2; ^§^ *P* < 0.01 100 mg/kg T2 versus 200 mg/kg T2; ^#^ *P* < 0.0001 200 mg/kg T1 versus 200 mg/kg T2.

In GT treated samples with 200 mg/kg dose a significant increase in IFN-*γ* secretion was detected in comparison to all samples, except for T2 controls (Figure 5(a)).

At T2, spleen IFN-*γ* release significantly increased in treated samples in comparison to the remaining samples. In addition, at T2, values were higher with 200 mg/kg dose in comparison to 100 mg/kg dose. (Figure 5(b)).

### 3.1. Basic Histology and Histochemistry

**Figure 6:**
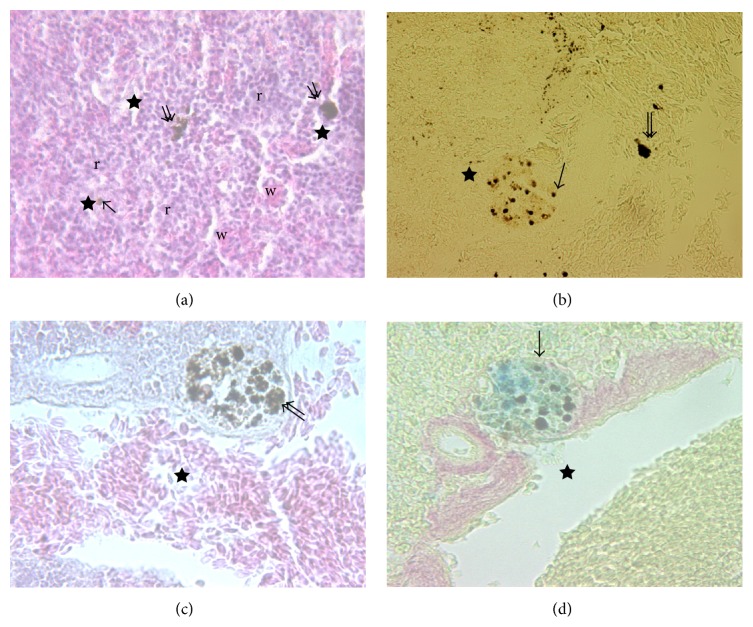
(a) Splenic tissue of* Dicentrarchus labrax* L. organized in areas of red pulp (r) and white pulp (w); the last consisting of ellipsoids, MØ (arrow), and MMC (double arrow) (H&E, 100x). ((b), (c), (d)) At higher magnification (400x) MØ and MMC appear distributed above all near vessels (star) (Anae, Mallory, and Perls-Van Gieson, resp.).

The histological appearance of spleen, stained with H&E, is shown in Figure 6(a).

These images show an outer capsule, pink stained, consisting of connective tissue and small trabeculae extended into the parenchyma, which can be divided into a red and white pulp, respectively. However, this arrangement is not in an orderly manner, as can be observed in spleen of higher vertebrates since the two types of tissue are always intermixed. Anyway, the red pulp consists of a reticular cell network supporting blood-filled sinusoids that hold diverse cell population, including MØ and lymphocytes, while the white pulp is composed of small spherical corpuscles also called “ellipsoids” [4], MØ free, and MMCs. Splenic ellipsoids are divided into arterioles forming dense-walled capillaries that are capable of collecting enormous quantities of small particulate antigens. MØ and MMCs exhibit irregular boundaries with densely filled cytoplasmic granules and other unidentified substances. Of note, it becomes difficult to distinguish the nucleus from the rest of the cytoplasm. For this reason, they were cytochemically identified with Perox and Anae staining (Figure 6(b)). The presence of ferric iron and lipofuscin-ceroids in MMCs was detected as dark brown granules with Mallory and Perls-Van Gieson staining, respectively (Figures 6(c) and 6(d)).

### 3.2. Quantitative Analyses of MMC

The size of MMCs varied greatly within the same section; some formed large clusters measuring up to 77 *μ*m in area, while others were smaller, less than 20 *μ*m, likely monocellular MØ. The single size of MØ and MMCs did not significantly differ between groups. Instead, number and sum of surfaces occupied by MØ and MMC were different in spleen samples from all farmed fish at T1 and at T2, respectively.

MØ percentage was higher in controls than that observed in treated fish at T1 with both polyphenol doses.

MMC percentage was lower in controls than that detected in treated fish with 200 mg/kg dose.

**Figure 7:**
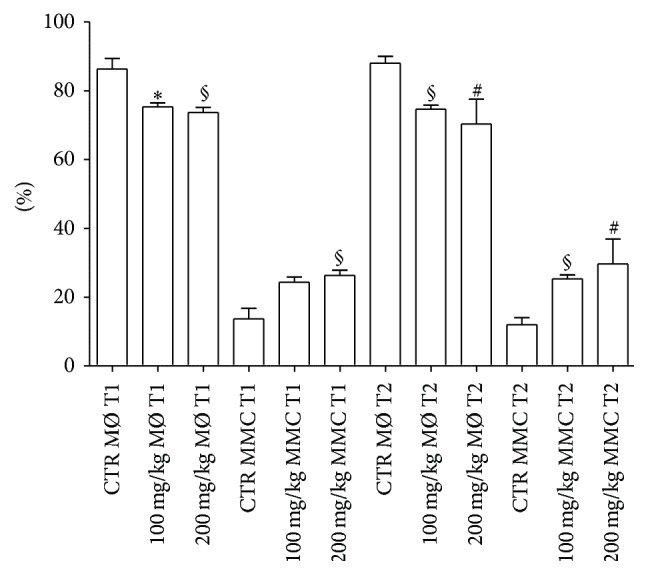
Spleen number percentages of MØ and MMCs from treated and untreated fish. For abbreviations and statistical analysis see Figure 1. ^*∗*^ *P* < 0.05 CTR MØ T1 versus 100 mg/kg MØ T1; ^§^ *P* < 0.01 CTR MØ T1 versus 200 mg/kg MØ T1; ^§^ *P* < 0.01 CTR MMC T1 versus 200 mg/kg MMC T1; ^§^ *P* < 0.01 CTR MØ T2 versus 100 mg/kg MØ T2; ^#^ *P* < 0.0001 CTR MØ T2 versus 200 mg/kg MØ T2; ^§^ *P* < 0.01 CTR MMC T2 versus 100 mg/kg MMC T2; ^#^ *P* < 0.0001 CTR MMC T2 versus 200 mg/kg MMC T2.

At T2, MMC percentage significantly increased with both concentrations of polyphenols in comparison to untreated fish (Figure 7).

**Figure 8:**
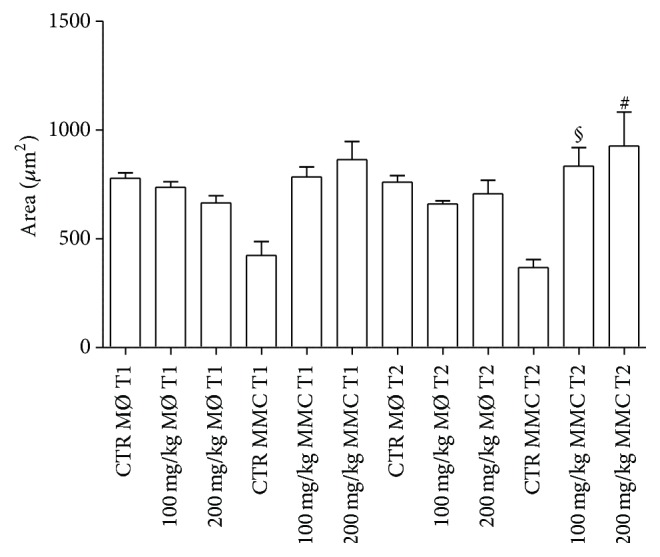
Spleen areas occupied by MØ and MMCs of treated and untreated fish. For abbreviations and statistical analysis see Figure 1. ^§^ *P* < 0.01 CTR MMC T2 versus 100 mg/kg T2; ^#^ *P* < 0.0001 CTR MMC T2 versus 200 mg/kg T2.

At T1 and T2, the area occupied by control MØ decreased compared to treated fish. On the contrary, the area occupied by MMCs increased in polyphenol-administered fish. In particular, MØ areas showed no statistical difference in controls versus treated fish at T1 and at T2. Instead, MMC areas were higher in treated fish when compared to controls at T2 with both polyphenol concentrations (Figure 8).

## 4. Discussion

Polyphenols are endowed with antioxidant and anti-inflammatory activities as documented by a series of data obtained in animals and humans [55–59]. Stemming from the concept that farmed fish are exposed to a continuous antigenic pressure owing to intensive rearing conditions in aquaculture, a robust stimulation of their immune system likely takes place. In principle, microbial challenges along with environmental insults (e.g., ultraviolet radiation) may trigger an early protective immune response in fish which may be converted into a chronic inflammation in the presence of a persistent immune stimulation [60, 61]. Ultimately, this pathological condition may increase fish mortality in aquaculture and/or lower quality of meat in terms of dietary consumption.

In the light of these considerations, we have treated farmed sea bass with a polyphenol-enriched feed in order to evaluate putative modifications of intestinal and splenic immune responses.

Release of cytokines from gut and spleen of treated and untreated fish has been evaluated under conditions of short term (2 h) or long term culture (24 h). In fact, previously, it has been demonstrated that production of fish cytokines is different according to length of incubation time of immune cells [62].

**Figure 9:**
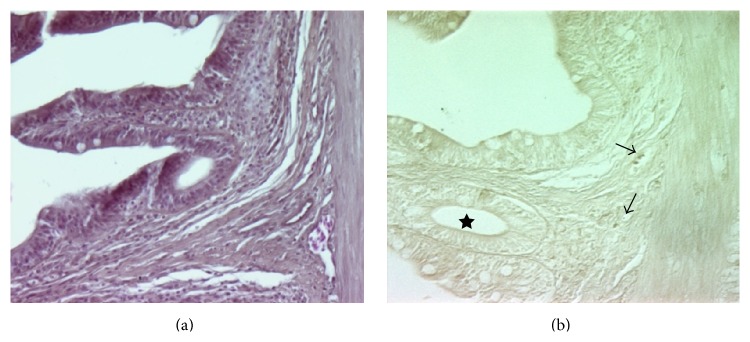
Intestinal sections from farmed sea bass. (a) (H&E staining, 250x) provides a general view of the gut. In (b) (Anae staining, 250x) a few lymphocytes (upper arrows) and one MØ (lower arrow) are evident in the lamina propria. Some blood vessels are present in the section (see star).

With regard to IL-1*β* concentrations, this cytokine decreases in treated samples in GP in comparison to controls after 2 h of incubation. In the spleen, no significant differences are observed between conventionally fed and polyphenol fed animals. It is well known that IL-1*β* is a proinflammatory cytokine characterized by the functional conservation of its signaling between mammalian and teleost lineages [63]. In principle, IL-1*β* protects the host against potential pathogens, while generating a detrimental inflammatory milieu in the case of its exaggerated production in response to persistent stimuli. As demonstrated by* in vitro* studies with sea bass, in head kidney leukocytes stimulated with* Vagococcus fluvialis* L-21 upregulation of IL-1*β* and Tumor Necrosis Factor (TNF)-*α* suggests an early inflammatory response [64]. This has also been confirmed by experiments in zebrafish following tissue injury [65]. In our experimental model, IL-1*β* reduction in treated samples seems to correlate with less fish mortality and reduced frequency of infections (manuscript in preparation), thus indicating a potential beneficial effect exerted by polyphenols. Quite interestingly, in our fish samples production of splenic IL-1*β* is much less than that observed in the intestines, thus suggesting a more effective capacity of spleen phagocytes to effectively destroy pathogens, thus leading to their immediate clearance. Instead, in the intestines the reduced contingent of MØ as well as lymphocytes, as documented by Figure 9(b), may lead to pathogen persistence with a continuous triggering of IL-1*β* release.

In mammals, IL-6 is an acute phase reactant which is also involved in hematopoiesis, inflammation, and immunomodulation, even including antibody production [66]. In fish, scarce information is available on the function of IL-6 despite the recent identification of its gene. In addition, IL-6 induced expression by IL-1*β* remains to be clarified in fish [67]. In our test system, decreases of IL-6 values occur in the GT (at T1 with 200 mg/kg dose/2 h) and in the spleen (at T1 with 200 mg/kg/24 h).

With regard to IFN-*γ* production, it is well known that this cytokine, mainly released by T helper (h)-1 cells and Th1-like cells [68, 69], is highly protective for the host against intracellular bacterial and viral infections. In our experiments, IFN-*γ* mostly increases in the spleen under the influence of polyphenols, thus indicating a more predominant immune adaptive function exerted by this lymphoid organ in comparison to the gut. Quite interestingly, in Atlantic halibut, experimentally infected with nodavirus, increased levels of T cell marker and IFN-*γ* transcripts have been observed as an example of a robust adaptive immune response. In the same context,* in vitro* experiments have also shown the intervention of IL-6 and IFN-*γ* with involvement of CD8*β*+ cells against nodavirus [70]. Furthermore, Jung and associates [71] have reported that recombinant IFN-*γ* protects the olive flounder (*Paralichthys olivaceus*) against* Edwardsiella* (*E.*)* tarda*, thus increasing survival in comparison to* E. tarda* treated group only.

In relevance to polyphenols used in our experiments, green tea administration [72] to rainbow trout (*Oncorhynchus mykiss*) was able to modulate immune-related gene expression of several cytokines, such as IL-1*β*, IL-6, IL-8, and IL-10 and TNF-*α* in spleen, liver, and kidney. In general terms, upregulation of all cytokines was observed except for the reduction of IL-10. Furthermore, an enhancement of the antioxidant system was demonstrated in green tea treated animals.

Finally, as far as polyphenol doses used in our feeds are concerned, no differences have been evidenced between 100 and 200 mg/kg concentrations used.

Fish MMCs are considered as an expression of primitive cellular aggregates where MØ engulf various pigments and cell debris but also exert an early protection against pathogens [16–18]. The analysis of MØ and MMCs demonstrates that treatment with polyphenols induces an increase of MMC numbers and related areas. This means that MØ may form clusters which generate MMCs which, in turn, may afford more protection to fish against pathogens, even including viruses. In fact, we can hypothesize that all trapped material is first engulfed by MØ and then transported to MMCs for their processing. Of note, it is possible to observe MMCs mainly located in close proximity of blood vessels. Reduction in MØ number by effect of polyphenols may explain the decrease of IL-1*β* while it is conceivable that MMCs may act as antigen presenting cells toward T lymphocytes, which in turn release IFN-*γ*. Therefore, increase in MMCs seems to contribute to less mortality and reduction of infectious events in farmed sea bass.

In conclusion, our results confirm and extend previous data related to the anti-inflammatory and immune-modulating activities exerted by polyphenols [45, 48, 59]. This contention is supported by the reduction of IL-1*β* and IL-6 concentrations and enhancement of spleen IFN-*γ* release. This dietary approach may be exploitable by farmed fish companies in terms of either longer fish survival or more beneficial effect for consumers owing to higher quality of meat.
